# Healthcare Professionals’ Subjective Well-Being: A Systematic Review and Methodological Appraisal of Conceptual Models, Measurement Instruments, and Associated Factors

**DOI:** 10.3390/ijerph23030329

**Published:** 2026-03-06

**Authors:** Iluta Skrūzkalne, Evija Nagle, Otto Andersen, Jeļena Perevozčikova, Luule Sakkeus, Antanas Kairys, Ingūna Griškēviča, Silva Seņkāne, Andrejs Ivanovs, Ieva Reine

**Affiliations:** 1 Statistics Unit, Rīga Stradiņš University, Dzirciema iela 16, LV-1007 Riga, Latvia; 2 School of Business and Law, University of Agder, Gimlemoen 25, 4630 Kristiansand, Norway; 3 Department of Demography, Tallinn University, Uus-Sadama 5, 10120 Tallinn, Estonia; 4 Faculty of Philosophy, Department of Psychology, Vilnius University, Universiteto g. 9, LT-01513 Vilnius, Lithuania; 5 Department of Health Psychology and Pedagogy, Rīga Stradiņš University, Dzirciema iela 16, LV-1007 Riga, Latvia; 6 Department of Public Health and Caring Sciences, Uppsala University, Husargatan 3, SE-751 22 Uppsala, Sweden

**Keywords:** healthcare professionals, subjective well-being, conceptual models, measurement instruments, influencing factors, systematic review

## Abstract

**Highlights:**

**Public health relevance—How does this work relate to a public health issue?**

- Healthcare professionals’ well-being has been reported in association with indicators related to patient safety, workforce sustainability, and healthcare system functioning.
- This systematic review synthesises current evidence on subjective well-being among healthcare professionals within a public health framework.

**Public health significance—Why is this work of significance to public health?**

- The findings demonstrate substantial conceptual and methodological heterogeneity in existing research on healthcare professionals’ well-being.
- Identifying conceptual models, research instruments, and examining determinants contributes to more structured population-level monitoring of workforce well-being.

**Public health implications—What are the key implications or messages for practitioners, policymakers and/or researchers in public health?**

- Multidimensional assessment frameworks may support the development of more coherent workforce policies and inform future research directions.
- Policymakers, healthcare managers, and researchers may use these findings to better understand factors associated with workforce sustainability in healthcare systems.

**Abstract:**

The well-being of healthcare professionals (HCPs) is widely recognised as a critical construct related to workforce sustainability, patient safety, and healthcare system performance; however, research in this area remains conceptually fragmented. This systematic review identifies and critically analyses conceptual models, assessment instruments, and factors associated with HCPs’ subjective well-being. A comprehensive literature search was conducted across six databases covering 2014 to 2024, focusing on quantitative empirical studies published in English in peer-reviewed journals. The review adhered to Preferred Reporting Items for Systematic Reviews and Meta-Analyses (PRISMA) guidelines. Study quality was assessed using the Joanna Briggs Institute criteria, and the methodological quality of measurement instruments was evaluated with the Consensus-based Standards for the Selection of Health Measurement Instruments (COSMIN) checklist in validation-focused studies. Of the 7838 records initially identified, 48 studies met the inclusion criteria. Three primary thematic areas emerged: (1) conceptual models framing subjective well-being, (2) measurement instruments assessing subjective well-being, and (3) factors associated with subjective well-being among HCPs. Frequently applied conceptual frameworks included the job demands–resources model, Maslach burnout theory, and WHOQOL-related approaches. Commonly used instruments comprised the WHO-5, Maslach Burnout Inventory, and Mini-Z. In validation-focused studies assessed using COSMIN criteria, internal consistency and aspects of construct validity were generally reported as acceptable; however, reporting across measurement property domains was variable. Factors examined in relation to subjective well-being included workload, emotional exhaustion, social support, autonomy, and work–life balance. Overall, the reviewed literature demonstrates substantial variability in conceptual and methodological approaches and frequently focuses on single dimensions of well-being. These findings highlight the potential value of developing integrated, sector-specific frameworks to inform future measurement development and research in this field.

## 1. Introduction

The scientific literature is characterised by a significant ambiguity in the definition of the concept of well-being, reflecting both different theoretical approaches and the focus of researchers on different aspects of well-being. Some authors conceptualise it as subjective well-being, including positive emotions, life satisfaction, and cognitive appraisal of life [1], while others view well-being more broadly as an individual’s psychological, emotional and social functioning in different life contexts, including professional settings [2,3]. This multiplicity of definitions makes comparability of empirical research findings difficult and points to the need for a common, domain-specific conceptualisation, particularly in studies of healthcare professionals. Throughout this review, the term “healthcare professionals (HCPs)” is used consistently to refer to licenced clinical staff working within healthcare settings.

The deterioration of the subjective well-being of healthcare professionals (SWHP) has increasingly been recognised as a global concern and a significant challenge within healthcare systems over the last fifteen years [4]. This trend was particularly exacerbated by the COVID-19 pandemic and global economic instability, which placed significant additional strain on both health systems and their staff [5]. Well-being in this context is understood as a multidimensional concept that encompasses emotional and psychological stability, physical health, and occupational functioning in the work environment [1,6].

In the present review, SWHP is conceptualised as the subjective evaluation of psychological balance, professional fulfilment, motivation, and perceived meaningfulness of work within the occupational context of HCPs. Deterioration in SWHP has been reported in association with indicators of staff resilience, professional sustainability, and perceived quality of patient care, underscoring its relevance within contemporary healthcare systems [4,7]. As defined by the World Health Organization (WHO), well-being encompasses optimal mental health, which is the ability to cope effectively with stressful situations, maintain high work productivity, and contribute positively to society [8]. In contrast, the National Academy of Medicine [NAM] in the United States of America defines well-being for HCPs as a combination of mental and physical health, the quality of which is significantly determined by the conditions of the work environment [6].

While these definitions provide a broader public health perspective, SWHP, as used in this review, refers specifically to HCPs self-perceived well-being in their professional role and should not be equated with general mental health status or the absence of psychological distress. SWHP includes personal perceptions of psychological balance, motivation, professional fulfilment, and meaningfulness of work in a specific healthcare setting. In contrast to generic or institutionally formulated definitions of well-being, SWHP focuses on the individual experiences of HCPs, which are considered important for professional sustainability and quality care [4,9].

Previous studies have reported associations between SWHP and indicators related to patient safety, as well as perceived aspects of healthcare quality [10]. At the individual level, low well-being has been found to co-occur with burnout syndrome and professional deformation [11], moral distress [12,13], and chronic stress [14,15,16]. Increased psychological distress, in turn, has been associated with reduced work capacity, lower motivation, and decreased professional effectiveness, including self-reported medical errors [17]. At the organisational level, lower levels of well-being among HCPs have been linked to high staff turnover, absenteeism, and reduced productivity. Persistent emotional and physical exhaustion has been reported in association with indicators of organisational strain and perceived reductions in service efficiency, which have been discussed in the literature in relation to patient safety and health system sustainability [7,9]. In addition, organisations experiencing high staff turnover may face increased financial demands, as recruitment and training of new professionals require additional resources [18].

Previous research on the subjective well-being of HCPs has often focused on single aspects of well-being, such as job demands, stress, or resources in the work environment [18,19]. In many cases, burnout, stress, or job demands have been examined as correlates or determinants of well-being, without clearly distinguishing these constructs from SWHP itself. While this approach provides important insights into specific risk factors, it only partially reveals the multidimensional nature of well-being. An integrated approach to fully understand and assess subjective well-being among HCPs is lacking.

Considering the current situation, there is a need to review and systematise recent studies using multidimensional, theoretically grounded conceptual models of subjective well-being and their corresponding assessment instruments. Such an approach would allow both the identification of the main influencing factors in different contexts and the assessment of the relevance of the most appropriate methodological approaches for assessing SWHP. This analysis aims to provide a clearer conceptual foundation for future empirical research and policy discussions concerning HCPs subjective well-being [5,20].

### 1.1. Aim

The aim of this systematic review was to comprehensively identify, critically appraise, and analyse empirically examined conceptual models of subjective well-being in healthcare professionals (SWHP) and their associated measurement tools, and to identify the most relevant statistically examined factors related to subjective well-being.

### 1.2. Research Questions

To achieve this objective, three research questions were set:

1. Which conceptual models explore the subjective well-being of HCPs?
2. What assessment tools are used to study the subjective well-being of HCPs?
3. What are the constituent factors of the subjective well-being of HCPs?

## 2. Research Methodology

To ensure structured and transparent data analysis and interpretation, the PRISMA (Preferred Reporting Items for Systematic Reviews and Meta-Analyses) guidelines were used to organise and report the results of this systematic review (see Supplementary Table S8: PRISMA 2020 Checklist). This approach supports a rigorous and reproducible review process, enabling systematic identification, selection, and synthesis of relevant studies. Adherence to PRISMA enhances the transparency and methodological consistency of the review, facilitating comparability across studies and reducing the influence of individual studies on the overall findings.

This systematic review was not prospectively registered in an international review registry (e.g., PROSPERO), and a formal review protocol was not published prior to conducting the research.

### 2.1. Search Strategy

Before conducting the systematic review, initial searches were conducted in October 2024 in the Cochrane Library and the Joanna Briggs Institute (JBI) database of systematic reviews and implementation reports, using keywords reflecting the focus of the review. Both generic terms such as “well-being” and more specific terms referring to the SWHP (e.g., “subjective well-being”, “healthcare professionals”, “clinician well-being”, “physician well-being”) were applied. The aim of this preliminary search was to examine existing systematic reviews and determine whether a review with a similar thematic focus had already been conducted. No systematic or scoping reviews were identified that met the objectives or thematic scope of the present review.

With the support of two librarians, a comprehensive search strategy was subsequently developed based on an initial rapid review of scientific literature. The search combined both Medical Subject Headings (MeSH) terms and free-text keywords commonly used in systematic reviews addressing the subjective well-being of HCPs and its associated factors. Boolean operators (AND, OR, NOT) were employed to combine and refine search terms, thereby improving search precision and ensuring the identification of relevant literature. The search strategy was tailored individually for each database platform, and the full search strings are provided in Supplementary Materials (Table S1).

A comprehensive literature search was conducted across six electronic databases (see Table 1). In addition, reference lists of all included studies were manually screened to identify potentially relevant articles not captured through the database searches. The search was limited to full text, peer-reviewed research articles published in English between 2014 and 2024.

The restriction to the period 2014–2024 was applied to ensure the inclusion of contemporary research reflecting recent developments in healthcare systems, workforce conditions, and well-being conceptualisations, particularly considering structural healthcare reforms and the impact of the COVID-19 pandemic. Earlier studies were not excluded due to lack of relevance, but to ensure a focused synthesis of contemporary empirical evidence.

The literature search was carried out between October 2024 and December 2024.

### 2.2. Study Selection Process

An iterative approach was applied during the initial article search to optimise sensitivity and relevance through pilot testing of search outputs [21]. Refinement of the search strategy was conducted prior to the formal screening process with minor adjustments to search terms and Boolean operators. These adjustments did not affect the predefined inclusion and exclusion criteria, which remained unchanged throughout the review process. To ensure methodological consistency and the relevance of the studies to the systematic review, study selection was based on predefined inclusion and exclusion criteria (see Table 2).

Quantitative empirical studies were included to ensure methodological homogeneity and allow systematic comparison of conceptual models, measurement instruments, and statistically examined factors related to subjective well-being. This focus enabled a structured synthesis of operationalised constructs and reported psychometric properties across studies.

Qualitative studies were excluded not due to a lack of relevance but rather to maintain comparability of empirical findings within a single methodological framework. While qualitative research contributes substantially to conceptual development and contextual understanding, the present review aimed specifically to synthesise quantitatively operationalised evidence.

Although qualitative research plays a central role in the development and theoretical refinement of conceptual models, the present review focused specifically on quantitatively operationalised and empirically tested models to enable structured comparison of constructs, measurement properties, and associated factors within a homogeneous methodological framework.

Review articles, commentaries, and theoretical papers were excluded because the objective of this review was to analyse primary empirical data rather than secondary interpretations of previously published findings.

### 2.3. Study Screening and Selection Process

After the search was completed and duplicates removed, all selected articles were imported into the reference management software EndNote 21. A two-stage screening procedure was implemented to ensure a systematic and reliable selection of studies. In the first stage, an initial selection was made based on article titles and abstracts. Attention was paid to identifying analytical observational (predominantly cross-sectional) and psychometric validation designs. Experimental and intervention studies, including randomised controlled trials, were excluded at this stage in accordance with the predefined eligibility criteria, as the review focused on conceptual models, determinants, and measurement instruments rather than intervention effectiveness.

Two independent investigators (EN and JP) assessed each study against the predefined inclusion and exclusion criteria. To enhance reliability, a third investigator (SS) independently reviewed a random 20% sample of excluded abstracts. In the second stage, a full text assessment was performed. All potentially eligible articles underwent full text eligibility assessment based on predefined criteria. The initial full text screening was carried out by EN, with particular attention to unclear cases, which were further discussed jointly with the other author during the data extraction stage. If there was uncertainty about the eligibility of an article, the final decision was made by discussion between at least two investigators until a consensus was reached. This approach ensured a common selection methodology and increased the reliability of the data analysis. Although formal interrater agreement statistics (e.g., Cohen’s κ) were not calculated, the screening process was conducted independently by two reviewers using predefined eligibility criteria. Discrepancies were systematically resolved through discussion and consensus, and a third reviewer independently assessed a random 20% sample of excluded abstracts to further enhance reliability and minimise selection bias.

**Figure 1:**
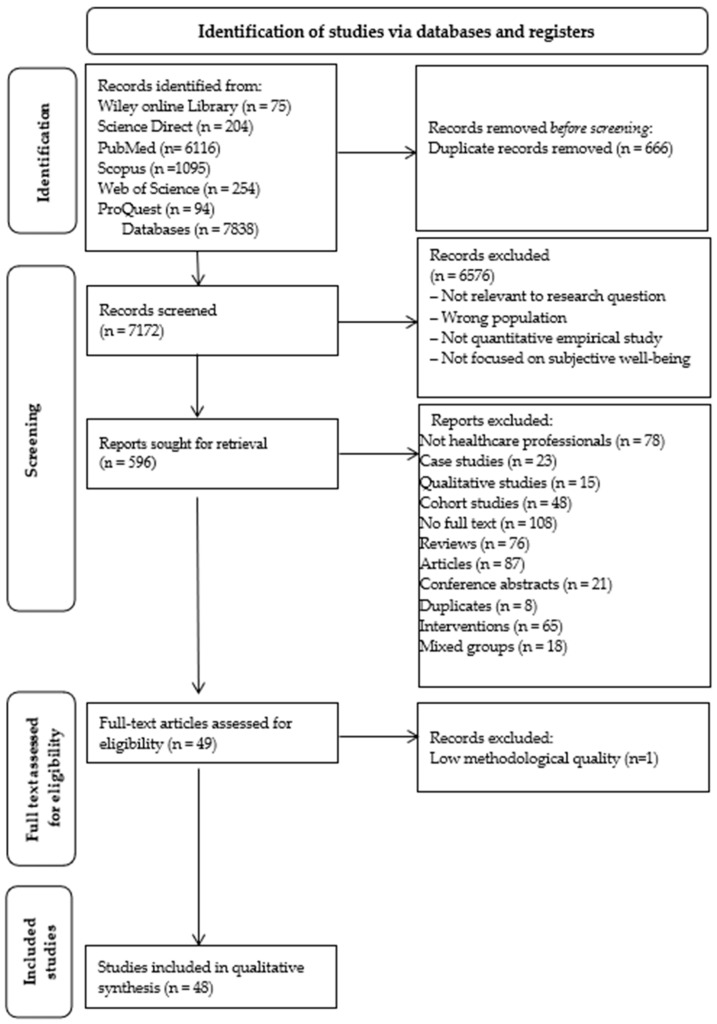
PRISMA flow diagram for systematic literature selection.

The study selection process was carefully documented in the PRISMA flow diagram (see Figure 1).

This structured selection process ensured a systematic and transparent identification of studies in accordance with predefined criteria, supporting the transparency and reproducibility of the review process.

### 2.4. Data Extraction

A special data synthesis table was developed to summarise the information from the studies. Initially created by researcher EN, the table was improved by IS and JP after testing with some studies. They collaboratively developed and refined the data extraction table using a shared Excel document, pilot testing it on a small sample of studies to ensure consistency and clarity in data collection.

Following pilot testing, operational definitions for each extraction category were specified to ensure conceptual clarity and consistency across reviewers. The extraction framework functioned as a structured coding scheme aligned with predefined research questions.

The table included the following data for each study:

- Bibliographic information and background data: author and year, country, study title and aim, design, population, and number of participants.
- Conceptual models and assessment tools for subjective well-being: conceptual models of subjective well-being identified in the literature and their corresponding assessment tools in the context of research on HCPs.
- Constituent factors: key aspects that contribute to the subjective well-being of HCPs.
- Methods of analysis: what methods were used to process the data.
- Results: main findings and conclusions.

Discrepancies were documented and resolved through structured comparison against the predefined coding framework. When consensus was not immediately achieved, a third investigator (SS) was consulted to adjudicate decisions. All coding decisions were documented within the shared extraction matrix to ensure transparency and traceability of the synthesis process.

### 2.5. Data Synthesis and Analysis Process

Thematic synthesis was conducted collaboratively by the research team. EN performed the initial structuring of findings, and the thematic framework was refined and validated through iterative discussion with IS, SS, IR, and AA. The synthesis followed a primarily deductive approach guided by the predefined research questions, while allowing for the identification of additional emergent themes where relevant. Initial codes were derived from the structured data extraction framework and subsequently grouped into higher-order thematic categories.

The data were analysed and synthesised following a three-stage approach:

- Data collection and descriptive analysis—initial structuring, identification of key outcomes.
- Thematic analysis and synthesis of results—identification and conceptual grouping of recurring themes.
- Interpretation of results and their implications for research and policy, including their relevance to the healthcare context.

Thematic categories were iteratively reviewed against the extracted data to ensure conceptual coherence and internal consistency. Refinement of themes was conducted iteratively until consensus was reached among the research team. Descriptive statistics and thematic synthesis of quantitative findings were conducted in alignment with the predefined data extraction and coding framework. All thematic decisions were documented within the shared synthesis matrix to ensure transparency and traceability of the analytical process. The results were interpreted in the light of the research objectives and questions, with consideration of their implications for future research and potential relevance for policy development.

### 2.6. Quality Appraisal and Bias Assessment

In the final phase of the screening, a quality assessment of the included studies was conducted to ensure methodological rigour and reliability of the findings. The JBI Critical Appraisal Checklist for Analytical Cross-Sectional Studies was applied to 41 empirical observational studies examining factors influencing HCPs’ subjective well-being [22]. This tool provides a structured evaluation of methodological quality and risk of bias (see Table 3).

Eight studies primarily focused on instrument development or validation and were therefore assessed using the COSMIN checklist (see Supplementary Materials Table S4), which is specifically designed for evaluating the methodological quality of studies on measurement properties.

Before assessing the quality of the studies, the research team carefully consulted the guidelines developed by the Joanna Briggs Institute (JBI) to ensure a methodologically sound and consistent approach to the assessment of studies. Each criterion was assessed using a response scale (‘Yes’, ‘No’, ‘Unclear’, or ‘Not applicable’) to objectively determine whether a particular study met the quality standards. As the JBI guidelines do not provide a strict scoring system for the overall quality of studies, each criterion was analysed individually, paying particular attention to methodological weaknesses and their potential impact on the reliability of the results. The risk of bias was assessed by two independent researchers (EN and JP), with each assessment conducted individually. The quality assessments provided by the two researchers were then compared and analysed. In cases where differences in assessment arose, a third investigator was recruited and the study in question was re-examined in detail until a final assessment was agreed. The final decision on whether to include a study in the systematic review was based on a detailed methodological analysis and an assessment of the potential risk of bias. In addition, to ensure a more structured and consistent interpretation, the research team developed an additional classification system to systematically identify studies of high, medium, or low methodological quality. To ensure a transparent and reproducible assessment process, the following classification of quality and risk of bias was used (see Table 4). Studies scoring ≥4 affirmative responses on the JBI critical appraisal checklist were eligible for inclusion.

While domain-level assessments remained central to the methodological evaluation, the structured categorisation supported transparent inclusion decisions and interpretation of overall study rigour. Prior to study selection, a minimum threshold of ≥4 affirmative responses on the JBI critical appraisal checklist were defined as a priori for study inclusion. Studies scoring ≤3 were excluded due to substantial methodological limitations and high risk of bias. The affirmative response count was applied as a structured decision-support criterion and did not replace domain-level methodological judgement. Studies presenting critical methodological limitations in key domains (e.g., confounding control or high risk of bias in core criteria) were evaluated individually, irrespective of the total number of affirmative responses.

This structured classification approach maintains the flexibility of the JBI guidelines while providing a clear decision-making framework that allows for systematic assessment of studies for inclusion in systematic reviews and meta-analyses. Any disagreements that arose were resolved through discussion and consensus.

Instrument reliability and validation studies were assessed using the COSMIN checklist (Consensus-based Standards for the Selection of Health Measurement Instruments) [23]. COSMIN is an internationally recognised framework developed through a Delphi consensus process and extensively applied in healthcare, psychology, and social science research [23,24].

The COSMIN framework comprises nine measurement property domains: content validity, structural validity, internal consistency, cross-cultural validity, reliability (test–retest), measurement error, hypothesis testing (construct validity), criterion validity, and responsiveness. Each property was evaluated according to predefined methodological standards based on data reported in the included studies. Two independent reviewers assessed the methodological quality of each study. Discrepancies were resolved through discussion to reach consensus. If consensus was not achieved, a third researcher with expertise in psychometric evaluation assigned the final rating. A researcher with expertise in psychometric evaluation assigned the final rating. Each measurement property received a qualitative rating following COSMIN recommendations: sufficient (+), insufficient (–), indeterminate (?) or not evaluated (NE) [23] (see Supplementary Materials Table S4).

COSMIN was selected as the primary appraisal tool due to its structured, transparent, and internationally accepted methodology for evaluating psychometric instruments. However, COSMIN ratings depend on the completeness and quality of reporting in primary studies, which may limit the certainty of some evaluations. This limitation was considered when interpreting the results.

## 3. Results

### 3.1. Search Method

A systematic literature search was conducted in six major academic databases: the Wiley Online Library, Science Direct, PubMed, Scopus, Web of Science, and ProQuest to identify studies on the subjective well-being of healthcare professionals (SWHP), conceptual models, research instruments and constituent factors of SWHP. A total of 7838 records were identified. Before the initial selection, 666 duplicates were removed, leaving 7172 records for further screening. At the screening stage, 6576 records that did not meet the inclusion criteria were excluded by analysing titles and abstracts. A total of 596 studies were selected for in-depth analysis and their full texts were carefully assessed. Of the studies initially identified, 547 were excluded based on the following exclusion criteria: not HCPs (*n* = 78), case study design (*n* = 23), qualitative research methodology (*n* = 15), cohort studies (*n* = 48), non-availability of full text (*n* = 108), literature reviews (*n* = 76), articles that did not meet the inclusion criteria (*n* = 87; e.g., studies focusing on general or objective well-being indicators), conference abstracts (*n* = 21), duplicates (*n* = 8), intervention studies (*n* = 65), and studies with heterogeneous participant groups (*n* = 17). In addition, an assessment of the methodological quality of the studies was performed. Consequently, 49 full text articles were assessed for eligibility. After methodological quality appraisal, one study (*n* = 1) was excluded due to low methodological quality, resulting in 48 studies being included in the final qualitative synthesis (see Figure 1).

### 3.2. Study Characteristics

A data synthesis table was created to analyse the included studies (see Supplementary Materials Table S2). The number of studies was relatively low and stable between 2015 and 2019, ranging between two and four studies per year. A notable increase was observed between 2020 and 2021 (nine studies each year).

In 2022, the number of studies decreased slightly (eight studies) but remained at a high level. In 2023 and 2024, there was a decrease in the number of studies, but the number of studies was still relatively high (four and six studies, respectively) compared to the pre-2020 period.

Most studies have been conducted in China and the USA. In Canada, four studies were identified, and Brazil, Spain, and Belgium had three studies each. Germany, Singapore, and the UK conducted two studies each. The remaining countries each conducted one study, including Finland, India, Nigeria, Saudi Arabia, Israel, Croatia, Czech Republic, Italy, Taiwan, Malta, Pakistan, Romania, South Korea, Australia, Slovenia, Ireland, Greece and Mexico (see Table 5).

The studies analysed identified different groups of clinicians used as samples for the subjective well-being study. The most common study samples were:

- Nurses—22 studies were included, including specific subgroup such as surgical nurses, mental health nurses, and nurses in hospital internal medicine and surgery departments.
- Doctors—identified in 20 studies. This group includes:
  ✓ Doctors in general, often referred to as “doctors” or “physicians”;
  ✓ Emergency physicians;
  ✓ Family physicians—mentioned as a separate professional group in three studies;
  ✓ Residents/interns—mentioned in two studies as doctors in training.
- HCPs—referred to in 10 studies under the umbrella term “healthcare professionals”, often without identifying specific professions.

In some studies, samples were drawn from mixed groups of professionals, such as doctors and nurses together, or the generic term ‘healthcare professionals’ was used. Each study was classified according to the primary professional group explicitly analysed. In cases where mixed samples (e.g., nurses and doctors combined) were examined without subgroup-specific analysis, studies were categorised under “healthcare professionals” to avoid double-counting. Each study was counted only once within the professional classification framework.

**Figure 2:**
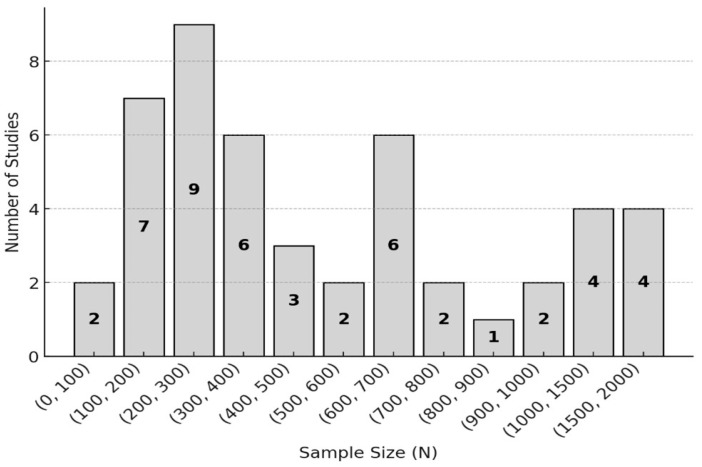
Distribution of study sample sizes. Numbers inside the bars indicate the number of studies within each sample size category.

The number of respondents in the surveys ranged from 200 to 500, reflecting predominantly medium-sized samples. Larger samples of more than 500 respondents were used less frequently, while smaller samples (fewer than 200 participants) were observed in a limited number of studies (see Figure 2). However, formal statistical power analyses were inconsistently reported, and the generalisability of the findings depended on study-specific sampling strategies and response rates. This distribution of sample sizes indicates methodological diversity and variation in study design rather than uniform representativeness.

Of 48 studies, gender was explicitly stated in 45. Most included studies had predominantly female samples (e.g., 3676 women and 353 men in one study; 1507 women and 208 men in another). Some studies had a more balanced gender distribution (e.g., 101 women and ninety-six men), while in three cases, gender was not indicated.

### 3.3. Quality and Risk of Bias in Included Studies

All 48 studies included in the final qualitative synthesis underwent methodological appraisal. Of these, 41 analytical cross-sectional studies were assessed using the Joanna Briggs Institute (JBI) Critical Appraisal Checklist for Analytical Cross-Sectional Studies [22], while the remaining seven studies focused primarily on instrument development and psychometric validation and were therefore evaluated using the COSMIN criteria [23,24], which are specifically designed for the appraisal of measurement properties.

The JBI Critical Appraisal Checklist for Analytical Cross-Sectional Studies, comprising eight domains, was applied in accordance with official guidance (see Supplementary Materials Table S3).

Among the 41 analytical cross-sectional studies appraised using the JBI checklist, 40 met the predefined threshold for high methodological quality according to the JBI criteria, and one met the threshold for moderate quality. None met the threshold for low methodological quality. Based on the predefined JBI appraisal domains, the overall risk of bias was considered low in 40 of these 41 studies and possible in one study. Although most studies met the predefined JBI criteria for high methodological quality, it should be noted that cross-sectional survey designs inherently carry limitations, including potential self-report bias, residual confounding, and limited causal inference. Therefore, the “high quality” classification reflects adherence to predefined methodological appraisal criteria rather than the complete absence of potential sources of bias. These findings should be interpreted with appropriate methodological caution.

In addition, the appropriateness and reliability of psychometric instruments were analysed using the COSMIN (Consensus-based Standards for the Selection of Health Measurement Instruments) criteria [23,74]. COSMIN appraisal was applied exclusively to studies in which instrument development or psychometric validation was the primary objective. This approach was necessary because COSMIN requires detailed reporting of measurement properties, which is typically available only in validation-focused studies.

Studies that merely applied previously validated instruments without reassessing their psychometric properties were not evaluated using the COSMIN framework, as the required methodological information was not reported. However, all instruments used across the included studies are listed in Supplementary Materials Table S6 to ensure transparency.

For validation studies meeting the eligibility criteria, the predefined COSMIN measurement property domains were systematically evaluated to assess the methodological quality and appropriateness of the instruments (see Supplementary Materials Table S4).

Of the full text articles assessed for eligibility (n = 49), eight studies focused on instrument development or psychometric validation and were evaluated using COSMIN criteria. Following domain-specific methodological appraisal, one validation study was excluded due to insufficient methodological quality across key measurement property domains (see Supplementary Materials Table S4). Consequently, seven validation studies were retained for the final COSMIN analysis, contributing to the total of 48 included studies. The domain-specific COSMIN appraisal demonstrated variability across measurement properties.

Internal consistency was the most consistently supported domain. All included validation studies demonstrated sufficient methodological quality in this domain, indicating adequate internal coherence of scale items.

Structural validity was rated as sufficient in most studies, suggesting that factor structures were appropriately tested and reported using acceptable methodological procedures.

Content validity showed greater heterogeneity. In several studies, the ratings were classified as indeterminate due to limited reporting on item development procedures, expert involvement, or theoretical justification.

Construct validity, assessed through hypothesis testing, was generally supported. Most studies demonstrated theoretical alignment between the instrument and the intended construct. However, in some cases, reporting limitations restricted the strength of conclusions.

Cross-cultural validity was inconsistently addressed. Only a subset of studies conducted formal adaptation procedures or measurement invariance testing. In several cases, insufficient information led to indeterminate ratings.

Reliability, particularly test–retest reliability, showed variability across studies. While some studies reported sufficient stability coefficients, others lacked detailed reporting regarding time intervals or reliability statistics, resulting in indeterminate ratings.

Criterion validity was adequately addressed in some studies, but in others, the absence of a clearly defined comparison standard or insufficient methodological detail limited evaluation.

Measurement error was not assessed in any of the included validation studies and was therefore classified as not evaluated.

Responsiveness was not assessed because the included studies used cross-sectional designs and did not report longitudinal data required to evaluate sensitivity to change. In accordance with COSMIN guidance, responsiveness was classified as not evaluated.

Overall, the findings indicate that internal consistency, structural validity, and construct validity were the most consistently addressed domains. In contrast, measurement error, responsiveness, and cross-cultural validity remain underrepresented and require more systematic evaluation in future validation research.

These results suggest that while several instruments demonstrate adequate methodological quality in specific measurement property domains, comprehensive validation across all COSMIN domains remains limited. Strengthening future validation procedures, particularly in underreported domains, would enhance measurement precision and improve the robustness and generalisability of findings in health and psychology research.

Three main thematic strands were identified and analysed about the subjective well-being of healthcare professionals (SWHP) as part of this systematic review:

**Theme 1:**  *Conceptual models used to investigate the subjective well-being of healthcare professionals.*

A detailed and systematic synthesis of the literature review data was carried out to identify the conceptual frameworks used to explore the subjective well-being of HCPs (see Supplementary Materials Table S5). Analysis of the results revealed that researchers use several theoretical approaches to understand the mechanisms and determinants of well-being in this specific professional group.

Overall, the analysis of the conceptual models shows that a variety of theoretical approaches, covering individual, organisational, and psychosocial aspects, are used to explore the subjective well-being of HCPs. All the models listed are based on studies that aimed specifically to investigate the subjective well-being of HCPs. These models differ in their methodological focus: some explain well-being as the outcome of a balance between workload and resources [75,76], others emphasise mechanisms of psychological resilience, motivation, and burnout [77]. The SWHP has been viewed as a multidimensional construct that integrates emotional, cognitive, and professional aspects, determining their emotional stability and professional sustainability [78,79]. The synthesis of different conceptual models provides a holistic understanding of the mechanisms of well-being and offers theoretically grounded approaches for its improvement in the healthcare sector.

**Theme 2:**  *Research tools used to assess SWHP.*

The assessment of the subjective well-being of healthcare professionals (SWHP) is an essential prerequisite for assessing the quality of life of HCPs, the characteristics of the work environment, the degree of professional burnout, as well as the general psychological and emotional state of HCPs. The results of such assessments may provide foundational evidence to inform the design of future intervention programmes [80]. To obtain accurate data on the well-being of HCPs, various psychometric tools are used to assess different psychological, social, and physical factors (see Supplementary Materials Table S6).

An assessment of the different tools used to assess subjective well-being reveals that the assessment of SWHP is a complex process that requires a multidimensional approach. As SWHP involves psychological, physical, and social factors, it is important to consider their interactions. Different factors such as emotional state, exhaustion, job satisfaction, social support, sleep quality, working conditions, and physical health are closely linked and influence each other. Effective assessment therefore requires instruments that cover a broader range of factors to obtain a complete picture of the well-being of SWHP [78,81]. As time goes by, the factors influencing SWHP change and so do the tools that need to adapt to today’s dynamic working and living environment. In recent years, the working conditions of HCPs have undergone significant changes, influenced by new factors such as technological developments, work flexibility, social distancing, and changes caused by pandemics. Widely used instruments such as the Maslach Burnout Inventory (MBI), Satisfaction with Life Scale (SWLS), and WHO-5 Well-Being Index remain psychometrically robust and widely applied; however, they were originally developed to assess specific dimensions of well-being and may not fully capture the broader occupational and contextual dimensions of SWHP in contemporary healthcare settings [78,82,83,84]. This observation reflects the evolving conceptualisation of SWHP as a multidimensional and occupationally embedded construct rather than a limitation of these established instruments. Importantly, these instruments remain psychometrically robust and valuable for assessing specific constructs; however, their combined or complementary use may be necessary to obtain a more comprehensive understanding of SWHP.

These findings suggest increasing interest in the development of multidimensional instruments that incorporate contextual and occupational factors relevant to contemporary healthcare settings. Such an approach may improve the comprehensiveness of assessment and may inform the development of targeted intervention strategies [76,85]. Integrating multi-instrument approaches may provide a more comprehensive and context-sensitive understanding of psychological, social, and occupational dimensions of well-being. Such insights could inform future intervention research rather than directly establishing predictive validity or intervention effectiveness [86]. Future instrument development would benefit from flexibility and adaptability to diverse working and cultural contexts. The tools must be able to account for specific sectoral and cultural characteristics that affect the working environment and the social environment of workers. This will increase the universality and practicality of the use of the tools, making them applicable in different organisations and work environments [87].

**Theme 3:**  *Factors associated with SWHP.*

The subjective well-being of HCPs has been widely recognised as an important construct associated with professional sustainability of HCPs and with indicators of patient care quality [20]. Given that most included studies employed cross-sectional designs, the identified factors should be interpreted as associated variables rather than established causal determinants [88]. The factors examined in relation to SWHP were divided into several categories, based on a multidimensional approach encompassing aspects examined in relation to SWHP rather than as inherent components of the construct itself. A detailed overview and references to studies analysing these factors are available in Supplementary Materials Table S7.

The categories were defined based on internationally recognised conceptual models and theoretical frameworks that explain the dynamics of the subjective well-being of HCPs, including the interaction between the work environment, and individual and social factors [89].

The factors examined in relation to subjective well-being were categorised as follows: Psychological factors encompass emotional, cognitive, and psychosocial dimensions that have been examined in relation to healthcare professionals’ subjective well-being, as mental health and emotional balance are essential for both professional effectiveness and long-term sustainability [90].

The most frequently examined psychological factors include depression (17 studies), anxiety (14), stress (11), and burnout (10). In the reviewed literature, these constructs were examined as factors associated with SWHP rather than as inherent components of subjective well-being. Additionally, life satisfaction (five studies) and general mental health (three studies) reflect the positive aspects of well-being and are also commonly explored. Less frequently studied, though still relevant, are psychological strengths and challenges such as mental resilience, compassion fatigue, positive emotions, psychological distress, and stress resilience. Each of these factors was examined in two studies. Furthermore, a range of additional individual-level factors, each investigated in a single study, has been identified across literature. Negative and positive psychological variables were examined as associated correlates in relation to reported SWHP, without being conceptualised as defining components of the construct.

Overall, the reviewed studies suggest that both negative and positive psychological variables are associated with variations in reported SWHP.

- Work environmental factors reflect structural and organisational aspects that can cause stress or contribute to a favourable working environment. This category includes workload, pay, job control, and resources, which have been examined in relation to occupational satisfaction and performance [91].

The working environment, including both physical and psychological conditions at the workplace, was the most frequently studied factor, with each aspect examined in eight individual studies. Following closely, workload, encompassing the intensity and quantity of tasks, was analysed in five separate studies, each focusing on its impact on stress and performance. Other work environment-related factors such as work involvement, work–life balance, job satisfaction, work control, job demands, and exposure to infectious diseases were each examined in four individual studies, underlining their importance in shaping healthcare professionals’ occupational experiences. Organisational and structural aspects, including management support, motivation, opportunities for professional development, communication with management, and patient mortality were each investigated in two separate studies, emphasising the role of leadership and systemic dynamics in healthcare settings. Additional workplace-related variables each assessed in a single study included feedback, autonomy, remuneration, meaningful work, availability of work resources, communication with colleagues, willingness to work in the healthcare sector, time constraints for task completion, and exposure to verbal and physical violence. Although these factors were less frequently explored, they reflect important dimensions of the occupational environment that may affect well-being, engagement, and retention.

In summary, the diversity and scope of workplace-related factors reflect the complex and multifaceted nature of healthcare settings. These structural and organisational variables have been associated with differences in reported emotional well-being and occupational experiences.

- Individual factors include biological and behavioural traits that influence work ability and well-being, such as sleep quality, subjectively perceived physical health, and nutrition [92]. In this review, physical health was considered only in terms of self-reported or perceived health status, rather than objective clinical or biomarker-based indicators. These characteristics may be associated with individuals’ capacity to cope with occupational demands. Health- and lifestyle-related factors were most frequently examined within this category. Sleep quality and physical health were each assessed in four studies. Nutrition, fatigue, and quality of life were each investigated in two studies, emphasising their influence on energy levels, emotional resilience, and overall well-being. In addition, several other health- and lifestyle-related factors were identified, with each examined in one respective individual study. These included physical activity (one study), self-rate well-being (one), loss of pleasure in daily activities (one), alcohol and tobacco use (one), meeting basic needs (one), and personality traits (one). Although less frequently explored, these factors provide meaningful insights into the broader factors associated with SWBHP.

Individual factors, including health and lifestyle aspects, form a critical foundation for understanding healthcare professionals’ subjective well-being, highlighting the need for a holistic approach in both research and support strategies.

- Social factors encompass interpersonal relationships and various forms of social support, which play a vital role in emotional stability and coping with stress [93]. These include not only general emotional and instrumental support but also more specific support networks such as family, workplace inclusion, and societal perceptions of health workers.

The most frequently examined factor was social support, which encompasses emotional, informational, and instrumental assistance received from others, and was assessed in six studies. Support from family and friends was evaluated in one study, highlighting the role of close personal relationships in helping individuals cope with professional and personal challenges. Involvement in decision-making, reflecting the extent to which HCPs can participate in workplace decisions, was explored in one study, emphasising its influence on motivation and job satisfaction. Additionally, social stigma, including societal stereotypes and negative pressures directed at HCPs, was addressed in one study and shown to negatively affect self-esteem and well-being, especially during periods of heightened public pressure.

Overall, social connections and perceived support were reported as factors associated with variations in SWHP.

- Demographic factors refer to relatively fixed characteristics, such as age, gender, marital status, education, and work experience, which have been examined in relation to healthcare professionals’ sensitivity to stress and occupational well-being. Across the included studies, demographic variables were most treated as control variables or covariates in multivariable analyses, and less frequently examined as primary independent predictors of SWHP.

The most frequently examined demographic factors were age and sex, which include biological characteristics associated with health and subjective well-being. Marital status, which has been examined in relation to social support and work–life balance, was analysed in six studies. Similarly, education, referring to the level of formal training and its impact on career progression and satisfaction, was also examined in six studies. Specialty, meaning the specific medical or healthcare field in which the individual works, was investigated in five studies in relation to workload and psychosocial demands. Lastly, work experience in healthcare, which has been analysed in relation to burnout, stress tolerance, and professional competence, was explored in four studies. In most studies, demographic characteristics were included to adjust for potential confounding effects rather than to establish them as primary explanatory variables. Overall, the findings highlight the multidimensional nature of SWHP and the diversity of factors examined in relation to it. Future research would benefit from integrated models that simultaneously consider individual, organisational, and social domains when examining SWHP [49,54].

Longitudinal and intervention-based studies are needed to clarify the directionality of observed associations and to evaluate potential strategies aimed at supporting healthcare professionals’ well-being. However, further empirical research is required to determine whether such strategies are associated with measurable improvements in healthcare professionals’ well-being and related organisational outcomes [94].

## 4. Discussion

The aim of this systematic review was to comprehensively identify, assess and analyse conceptual models and measurement tools for subjective well-being in healthcare professionals (SWHP) and to identify the main influencing factors. The results were compared with existing literature to assess relevance and identify new research trends.

### 4.1. Conceptual Models for the Study of the Subjective Well-Being of Healthcare Professionals

Like previous studies [20,95], this review is dominated by workload and resource balance models, in particular, the job demands–resources model [75] and the job control model [91], which emphasise structural factors as important variables examined in relation to the subjective well-being (SWHP) of HCPs. These models are often used to explain the emergence of burnout and stress mechanisms in healthcare.

Similarly, Maslach’s burnout theory [96], which identifies emotional exhaustion, depersonalisation, and a low sense of professional efficacy, is consistent with risk factors identified in other studies [15,19]. These studies describe a common problem of burnout in healthcare. At the same time, this review reveals that researchers are also increasingly using positive psychology and motivational theories, such as self-determination theory [97] and the psychological well-being model [78]. These frameworks emphasise internal psychological resources, such as autonomy, competence, and meaningfulness, as variables examined in relation to job stress and well-being.

This diversity of approaches shows that SWHP is not seen as an isolated concept but is emerging as an integrated and multidimensional theoretical framework that combines both stress and burnout models and positive psychological perspectives. This enables the analysis of SWHP as a professionally specific phenomenon in which both individual and organisational factors are equally important. Systemic and holistic models identified in the review, such as the OECD Occupational Well-Being Model [98] and the Concept of Work Well-Being [99], are used less frequently but offer a holistic view including working conditions, development opportunities, social support, and work–life balance. This is in line with Brigham et al.’s [6] call for integrated measurement models, particularly in the context of healthcare post-COVID-19. Overall, the results reveal that while stress and burnout models dominate literature, there is an increasing focus on internal and organisational resources.

This diversity suggests the potential value of developing a coherent, multidimensional theoretical framework that conceptualises SWHP as a systemic and profession-specific phenomenon associated with both environmental and individual factors.

The results of the review indicate that SWHP has been conceptualised as an integrative perspective that extends beyond traditional models of stress and burnout while incorporating elements of positive psychology.

### 4.2. Tools for Studying the Well-Being of Healthcare Professionals

The wide range of psychometric instruments identified covers different psychological, emotional, social, and work environment aspects [100]. Like previous studies [78,81,101], this review is dominated by instruments developed for the general population rather than specifically for healthcare professionals. This may limit the extent to which such instruments capture the profession-specific and organisational characteristics of healthcare settings. Although tools such as the Maslach Burnout Inventory, Satisfaction with Life Scale, WHO-5 Well-Being Index, and depression and anxiety screening instruments (PHQ-9, GAD-7, DASS-21) are widely validated and effectively assess certain aspects of subjective well-being, they primarily assess specific dimensions of well-being and may not consistently provide an integrated, occupationally contextualised assessment of SWBHP. They may not fully capture specific factors such as workload, social support, work–life balance, and psychosocial risks inherent to the healthcare environment, such as moral distress [19,102]. Given the relevance of this phenomenon, in recent years there has been a growing interest in the adaptation and validation of moral distress instruments among HCPs in different countries [103,104], including Latvia [13]. These efforts demonstrate the need to broaden the scope of well-being measurement tools.

The Well-Being Index (WBI) exemplifies a multidimensional instrument specifically designed for healthcare professionals, integrating multiple domains of distress and well-being in a concise format [105]. Although not all WBI-related publications met the predefined inclusion criteria of this review, its conceptual framework offers valuable guidance for developing integrated, profession-specific assessment tools.

In contrast to previous studies, this review emphasised the importance of a multidimensional approach that integrates psychological, physical, and social aspects. A similar position is also expressed in the work of Trockel and colleagues [33], who point out that traditional tools such as the MBI primarily focus on specific constructs (e.g., burnout) and may not encompass emerging occupational stressors such as technological overload, work flexibility, or pandemic-related challenges [106].

Moreover, the results show that existing tools are fragmented in terms of topics, focusing on individual components such as burnout, stress, emotional state, or job satisfaction, but rarely covering all relevant dimensions of well-being in a single framework. This thematic fragmentation may reduce the likelihood that certain factors—such as resilience, sleep quality, social support, or meaningfulness of work—are assessed within a single conceptual framework. These observations are consistent with the findings of Smith & Jones [80] and the World Health Organization [85] that a flexible, culturally specific, and contextually applicable approach is needed to assess the subjective well-being of healthcare professionals. Differences in work organisation, occupational risks, and cultural contexts across countries and healthcare settings limit the applicability of universal tools. New multidimensional tools that integrate emotional, occupational, social, and organisational dimensions may therefore contribute to a more comprehensive conceptualisation of well-being. Such instruments may contribute to improving the comprehensiveness of assessment and facilitating the identification of risk-related variables relevant for intervention planning [76,107]. In addition, it is essential to ensure that assessment tools are adaptable to different groups of HCPs and diverse work contexts, accounting for variables such as gender, age, professional status, and cultural characteristics.

The development and implementation of multidimensional assessment tools in healthcare settings present practical challenges. HCPs frequently face high workloads and time constraints, which can reduce their willingness to complete lengthy surveys. Researchers often strive to balance comprehensiveness with brevity to minimise survey fatigue and decrease non-response rates. Consequently, developing multidimensional tools necessitates balancing conceptual completeness with practical feasibility. Given the multidimensional and profession-specific characteristics of SWHP, further development and empirical validation of targeted, multidimensional assessment tools appears to be warranted.

### 4.3. Constituent Factors of the Subjective Well-Being of Healthcare Professionals

The results of this systematic review provide a comprehensive insight into the factors influencing the subjective well-being of healthcare professionals (SWHP), supporting the view of SWHP as a multidimensional construct. Like previous studies [18,94], this analysis reveals that well-being has been examined in relation to individual, work environment, social, and demographic aspects, which may demonstrate interactive associations.

The risk factors most often studied and identified relate to the psychological dimension, including depression, anxiety, stress, and burnout. These indicators reflect similar trends in the previous literature, which has highlighted the central role of mental health in professional effectiveness and emotional resilience [77,90]. Structural factors of the work environment have been associated with variations in reported well-being [108]. Leadership behaviours have been identified in seminal research as a critical determinant of clinician burnout and professional satisfaction. Studies by Shanafelt and colleagues demonstrated that participatory and supportive leadership styles are strongly associated with lower burnout rates and improved well-being among physicians [15,18]. Although leadership was examined explicitly in only a limited number of studies in the present review, the broader literature consistently highlights its importance in healthcare settings.

Previous research, including Theorell [109] and Bakker & Demerouti [110], suggests that organisational conditions can serve as both resources and sources of stress. Individual factors such as sleep quality, physical and mental health, and lifestyle have been identified as important adaptive mechanisms that determine the ability of clinicians to maintain work capacity under stressful conditions. These results are in line with Walker’s [111] and Warburton et al.’s [112] findings on the importance of a healthy lifestyle and restorative sleep in occupational performance. Social support variables were reported as being associated with higher levels of well-being. These results are consistent with Cohen & Wills [113], who suggest that social support reduces the negative effects of stress on emotional stability. Workplaces characterised by cooperation, trust, and mutual support have been associated with lower reported levels of burnout and higher job satisfaction. Supportive and participatory leadership styles enhance this protective effect because leadership behaviours significantly impact workplace climate, perceived support, and clinician well-being (e.g., [15,114]. Finally, demographic factors, particularly age, gender, education, and work experience, were identified as important variables determining individual sensitivity to stress and professional challenges. These findings are in line with Dollard [115] and Tilden [116], who stress that younger and less experienced workers are at greater risk of experiencing occupational difficulties. A differentiated approach to well-being tailored to different groups of workers may be beneficial when designing sustainable workforce strategies.

Overall, the results of this review strengthen the understanding of SWHP as a multidimensional phenomenon influenced by interrelated psychological, social, individual, and structural factors. This supports the need for a holistic approach in both research and practice to ensure the well-being and sustainability of healthcare professionals.

### 4.4. Strengths and Limitations of This Study

This systematic review has several notable strengths. First, a comprehensive and structured search strategy was applied across six major international databases, complemented by manual reference screening, which reduced the likelihood of missing relevant studies. Second, the review followed the PRISMA guidelines, ensuring transparency and methodological rigour throughout the identification, selection, and reporting processes. Third, the inclusion of established quality appraisal frameworks, namely the Joanna Briggs Institute criteria for study quality assessment and the COSMIN checklist for evaluating measurement instruments, strengthened the robustness and credibility of the findings. In addition, the review provides an integrated overview by simultaneously examining conceptual models, assessment instruments, and determinants of healthcare professionals’ subjective well-being, offering a multidimensional perspective that is often lacking in previous reviews.

Despite these strengths, several limitations should be considered when interpreting the results. The included studies demonstrated substantial heterogeneity in conceptual frameworks, measurement instruments, and psychometric reporting, which limited the possibility of quantitative synthesis and direct comparability of findings. Furthermore, most studies focused on single dimensions of well-being, such as burnout or stress, rather than adopting a holistic, multidimensional approach, potentially underrepresenting the complexity of subjective well-being. Further limitation involves the reliance on quantitative self-reporting measures. While qualitative and mixed-methods research offers valuable contextual and experiential insights, these approaches were excluded from the present review due to predefined inclusion criteria emphasising quantitative empirical studies. Finally, the restriction to English-language, peer-reviewed publications published between 2014 and 2024 may have resulted in the exclusion of relevant studies from other languages or publication formats.

In addition, the exclusion of qualitative studies and secondary review literature may have limited the conceptual depth and theoretical integration of the findings. Although qualitative research often contributes to the foundational development and refinement of conceptual models, the present review prioritised quantitatively operationalised and empirically examined frameworks to ensure methodological comparability across studies. Systematic and narrative reviews have provided integrated multidimensional conceptual frameworks based on previously published primary studies that were excluded due to predefined eligibility criteria. While this decision was made to ensure methodological homogeneity and comparability of quantitative evidence, it may have resulted in the omission of important contextual and interpretative perspectives. Furthermore, although efforts were made to access full text articles through institutional databases and reasonable attempts to obtain unavailable manuscripts, it is possible that some relevant studies were not included due to accessibility constraints. In addition, formal interrater agreement statistics (e.g., Cohen’s κ) were not calculated during the screening stage, which may limit the ability to formally quantify reviewer consistency, despite the implementation of independent dual screening and structured consensus procedures. Finally, the predominance of cross-sectional designs among the included studies limits causal interpretation of the observed associations between examined factors and subjective well-being, and the findings should therefore be interpreted with appropriate caution.

### 4.5. Implications and Future Directions

The findings of this systematic review have several important implications for research, practice, and policy in the field of healthcare professionals’ subjective well-being. First, the identified conceptual fragmentation and the predominance of single-dimension outcome measures highlight the need for integrated, multidimensional frameworks that capture both individual and work-related components of well-being. Future research may benefit from moving beyond isolated indicators such as burnout or stress and adopting more holistic models that reflect the complex and dynamic nature of well-being within healthcare settings.

Second, the widespread use of self-report quantitative instruments underscores the importance of further strengthening measurement validity and contextual sensitivity. Future studies should prioritise rigorous instrument validation processes, including cross-cultural adaptation, measurement invariance testing, and longitudinal assessment, to improve the precision and comparability of findings across different healthcare systems and professional groups.

Third, incorporating qualitative and mixed-methods approaches may provide deeper insights into the contextual, organisational, and psychosocial mechanisms underlying subjective well-being. Such approaches could complement quantitative findings by capturing lived experiences, professional values, and systemic constraints that are often not fully represented in standardised instruments.

From a practical perspective, the results may provide a conceptual basis for the future development of system-level strategies, pending further longitudinal and intervention-based evidence. Policymakers and healthcare organisations may consider exploring the feasibility of integrating validated, multidimensional well-being assessments into workforce monitoring, acknowledging the current limitations of the available evidence base. Overall, future research may further explore and refine context-sensitive, theoretically grounded, and methodologically robust approaches.

## 5. Conclusions

The results of this systematic review indicate that research on subjective well-being in healthcare professionals (SWHP) has been conducted using a variety of conceptual models and psychometric instruments, many of which primarily assess specific dimensions of well-being rather than integrating multiple psychological, social, and occupational domains within a single framework. In this review, SWHP was conceptualised as a multidimensional construct encompassing psychological, social, individual, and work-environment domains, as reflected in the thematic synthesis.

Existing instruments are mostly developed for the general population without accounting for the specificities of the healthcare sector and often focus on narrow aspects such as burnout [77], stress [113], or life satisfaction [83], ignoring the important interrelationships between psychological, social, individual, and work environment factors. A similar fragmentation can be observed at the theoretical level; this concerns the conceptual models used, such as the job demands–resources model [75], or self-determination theory [97], which offer different but limited perspectives. Although these frameworks provide valuable insights into specific mechanisms, they do not offer an integrated understanding of SWHP as a single, multidimensional phenomenon.

Given these limitations, the findings suggest the potential value of further improving conceptual integration in the assessment of SWHP. This may involve the development of multidimensional instruments, the harmonisation of existing constructs, or the use of modular assessment approaches that capture complementary domains within a coherent framework. The development of such instruments may involve integrating different conceptual models into a more unified theoretical framework that allows the assessment not only of individual adaptability and psychological state but also of structural risk and protective factors such as work environment, management support, and professional development opportunities [110].

A holistic approach based on multidimensional theoretical integration may provide a more comprehensive understanding of the subjective well-being of SWHP. Such an approach may provide a theoretical foundation for future intervention-oriented research and policy discussions, subject to empirical validation. In this context, SWHP can be seen as an emerging integrative conceptual perspective that offers a holistic understanding of the professional, emotional, social, and organisational dimensions relevant to healthcare professionals. It integrates different factors and highlights the specificities of the profession, which have often been underrepresented in traditional research.

## Figures and Tables

**Table 1:** Electronic databases were used and number of results found.

Databases	The Number of Selected Scientific Articles
Wiley Online Library	75
Science Direct	204
Pubmed	6116
Scopus	1095
Web of Science	254
ProQuest	94

**Table 2:** Inclusion and exclusion criteria.

Inclusion Criteria	Exclusion Criteria
Study design	
Quantitative empirical studies (observational and psychometric validation studies) examining subjective well-being using operationalised measures.	Experimental and intervention studies (including randomised controlled trials).
Language	
English	Non-English articles
Full text availability	
Full text articles accessible through institutional databases or reasonable efforts to obtain the manuscript.	Articles for which full text could not be retrieved after reasonable attempts (e.g., institutional access, interlibrary loan, or author contact).
Quality of publication	
Articles published in peer-reviewed scientific journals.	Publications that are not peer-reviewed.
The focus of research on well-being	
Studies explicitly examining subjective well-being or its clearly defined dimensions.	Objective aspects of well-being are addressed.
Study population	
HCPs (nursing assistants were not included in the study).	Non-medical respondents.
Period of publication	
2014–2024 period.	Studies before 2014 and after 2024.
Study quality	
Studies scoring ≥4 affirmative responses on the JBI critical appraisal checklist, as predefined a priori, were eligible for inclusion.	Studies scoring ≤3 affirmative responses on the JBI checklist were excluded due to substantial methodological limitations and were therefore not eligible for inclusion.

**Table 3:** Assessment criteria for the quality of empirical research.

No.	Quality Criterion	Question-Wording
1	Quality of selection of participants	Does the study clearly define inclusion and exclusion criteria and is the sample representative of the target population?
2	Suitability of the study population	Is the study population appropriate for the research question and does it accurately reflect the phenomenon being analysed?
3	Accuracy of measurements	Were the independent variables assessed using valid and reliable methods?
4	Identification and control of confounding factors	Has the study identified potential confounding factors and have these been taken into account in the analysis?
5	Reliability of outcome variable measurements	Have valid and reliable instruments been used to assess the outcome variables?
6	Suitability of statistical analysis	Are the statistical methods used appropriate to the study design and were they applied correctly?
7	Generalisability of the study results	Are the results discussed in relation to their generalisability, and is the sample size reported and justified in the context of the study objectives?
8	Ethical considerations of the study	Did the research follow ethical guidelines, such as obtaining informed consent and protecting confidentiality?

**Table 4:** JBI methodological quality assessment of studies.

Number of ‘Yes’ Answers	Quality Level	Risk of Bias	Inclusion in the Systematic Review
≥6	High	Low	Included because methodologically rigorous and reliable
4–5	Medium	Possible	May be included but needs careful interpretation
≤3	Low	High	Not included due to methodological limitations

**Table 5:** Geographical distribution of studies.

Country	Count	Reference
China	5	[25,26,27,28,29]
USA	5	[30,31,32,33,34]
Canada	4	[35,36,37,38,39]
Brazil	3	[40,41,42]
Spain	3	[43,44,45]
Belgium	3	[46,47,48]
Germany	2	[49,50]
Singapore	2	[51,52]
United Kingdom	2	[53,54]
India	2	[55,56]
Finland	1	[57]
Nigeria	1	[58]
Saudi Arabia	1	[59]
Israel	1	[60]
Croatia	1	[61]
Czech Republic	1	[62]
Italy	1	[63]
Taiwan	1	[64]
Malta	1	[65]
Pakistan	1	[66]
Romania	1	[67]
South Korea	1	[68]
Australia	1	[69]
Slovenia	1	[70]
Ireland	1	[71]
Greece	1	[72]
Mexico	1	[73]

## Data Availability

The datasets and supplementary findings generated and analysed during this study are included in the Supplementary Materials attached to this article.

## Supplementary Materials

The following supporting information can be downloaded at https://www.mdpi.com/article/10.3390/ijerph23030329/s1: Table S1: Search strategy of all Databases; Table S2: Characteristics of the studies included; Table S3: Assessment of the methodological quality and risk of bias of the included studies based on the Joanna Briggs Institute (JBI) Critical Appraisal Checklist for Analytical Cross-Sectional Studies; Table S4: Assessment of the Appropriateness and Reliability of Psychometric Instruments Based on the COnsensus-based Standards for the Selection of Health Measurement Instruments (COSMIN) Checklist; Table S5: Conceptual models within which the well-being of healthcare professionals was studied; Table S6: Research Instruments Used in the Study of Healthcare Professionals’ Subjective Well-Being; Table S7: Factors influencing the subjective well-being of healthcare professionals; Table S8: PRISMA 2020 Checklist. References [117,118,119,120,121,122,123,124,125,126,127,128,129,130,131,132,133,134,135,136,137,138,139,140,141,142,143,144,145,146,147,148,149,150,151,152,153,154,155,156,157,158,159,160,161,162,163,164,165,166,167,168,169,170,171,172,173,174,175] are cited in the Supplementary Materials.
