# Supplementary material for: Healthcare Professionals’ Subjective Well-Being: A Systematic Review and Methodological Appraisal of Conceptual Models, Measurement Instruments, and Associated Factors
Source: Int J Environ Res Public Health. 2026 Mar 6;23(3):329. doi: 10.3390/ijerph23030329 (PMC13026122; doi:10.3390/ijerph23030329)
Supplement: Supplementary file 1 [file ijerph-23-00329-s001.zip › S1_Search strategy.pdf]

Table S1. Search strategy of all Databases.

Database	Search Procedure
Wiley online Library	“(Nurse OR Nurses OR “Healthcare personnel” OR “Medical personnel” OR Physician OR Physicians OR Doctor OR doctors OR “physician assistant” OR “physician assistants” OR Clinician OR clinicians OR “Medical staff” OR “Medical staffs” OR “Healthcare practitioner” OR “Healthcare practitioners” OR “Healthcare worker” OR “Healthcare workers” OR “Hospital staff” OR “Hospital staffs”) AND (Well-being OR wellbeing OR “Healthcare workload” OR “Mental health” OR prosperity OR “Worker health” OR “Quality of life” OR “Job satisfaction” OR “Psychological well-being” OR “Compassion satisfaction” OR Wellness OR Self-Care OR “Occupational health” OR “Work environment” OR “Mental stress” OR “Professional burnout” OR Anxiety OR Depression OR Burnout OR “Job stress” OR stress) AND (tool OR tools OR measurement OR measuring OR measurements OR Instrument OR Instruments)”
Science Direct	(Healthcare personnel OR Healthcare practitioner OR “Healthcare worker”) AND (Well-being OR wellbeing OR “Quality of life” OR “Job satisfaction”) AND (tool OR Instrument)
Pubmed	((((((((((((((((((((((((“Health Personnel”[Majr]) OR (Nurse[Title/Abstract])) OR (Nurses[Title/Abstract])) OR (“Healthcare personnel”[Title/Abstract])) OR (“Healthcare personnels”[Title/Abstract])) OR (“Medical personnel”[Title/Abstract])) OR (Physician[Title/Abstract])) OR (Physicians[Title/Abstract])) OR (Doctor[Title/Abstract])) OR (Doctors[Title/Abstract])) OR (“physician assistant”[Title/Abstract])) OR (“physician assistants”[Title/Abstract])) OR (Clinician[Title/Abstract])) OR (Clinicians[Title/Abstract])) OR (“Medical staff”[Title/Abstract])) OR (“Medical staffs”[Title/Abstract])) OR (“Healthcare practitioner”[Title/Abstract])) OR (“Healthcare practitioners”[Title/Abstract])) OR (“Healthcare worker”[Title/Abstract])) OR (“Healthcare workers”[Title/Abstract])) OR (“Hospital staff”[Title/Abstract])) OR (“Hospital staffs”[Title/Abstract])) OR (“Nurses”[Majr])) AND ((((((((((((((((“Psychological Well-Being”[Majr]) OR (Psychological well-being[Title/Abstract])) OR (“Compassion satisfaction”[Title/Abstract])) OR (Wellness[Title/Abstract])) OR (Self-Care[Title/Abstract])) OR (“Occupational health”[Title/Abstract])) OR (“Work environment”[Title/Abstract])) OR (“Mental stress”[Title/Abstract])) OR (“Professional burnout”[Title/Abstract])) OR (Anxiety[Title/Abstract])) OR (Depression[Title/Abstract])) OR (Burnout[Title/Abstract])) OR (“Job stress”[Title/Abstract])) OR (stress[Title/Abstract])) OR (“Burnout, Professional”[Majr])) OR (“Occupational Stress”[Majr]))) AND (tool[Title/Abstract] OR tools[Title/Abstract] OR measurement[Title/Abstract] OR measuring[Title/Abstract] OR measurements[Title/Abstract] OR Instrument[Title/Abstract] OR Instruments[Title/Abstract])
Scopus	(TITLE(nurse OR nurses OR “Healthcare personnel” OR “Medical personnel” OR physician OR physicians OR doctor OR doctors OR “physician assistant” OR “physician assistants” OR clinician OR clinicians OR “Medical staff” OR “Medical staffs” OR “Healthcare practitioner” OR “Healthcare practitioners” OR “Healthcare worker” OR “Healthcare workers” OR “Hospital staff” OR “Hospital staffs”) AND TITLE(well-being OR wellbeing OR “Healthcare workload” OR “Mental health” OR prosperity OR “Worker health” OR “Quality of life” OR “Job satisfaction” OR “Psychological well-being” OR “Compassion satisfaction” OR wellness OR self-care OR “Occupational health” OR “Work environment” OR “Mental stress” OR “Professional burnout” OR anxiety OR depression OR burnout OR “Job stress” OR stress) AND TITLE(tool OR tools OR measurement OR measuring OR measurements OR instrument OR instruments)) AND PUBYEAR > 2013 AND PUBYEAR < 2025 AND (LIMIT-TO (LANGUAGE,”English”))
Web of Science Core Collection	Nurse OR Nurses OR “Healthcare personnel” OR “Medical personnel” OR Physician OR Physicians OR Doctor OR doctors OR “physician assistant” OR “physician assistants” OR Clinician OR clinicians OR “Medical staff” OR “Medical staffs” OR “Healthcare practitioner” OR “Healthcare practitioners” OR “Healthcare worker” OR “Healthcare workers” OR “Hospital staff” OR “Hospital staffs” (Title) and Well-being OR wellbeing OR “Healthcare workload” OR “Mental health” OR prosperity OR “Worker health” OR “Quality of life” OR “Job satisfaction” OR “Psychological well-being” OR “Compassion satisfaction” OR Wellness OR Self-Care OR “Occupational health” OR “Work environment” OR “Mental stress” OR “Professional burnout” OR Anxiety OR Depression OR Burnout OR “Job stress” OR stress (Title) and tool OR tools OR measurement OR measuring OR measurements OR Instrument OR Instruments
ProQuest	(Nurse OR Nurses OR “Healthcare personnel” OR “Medical personnel” OR Physician OR Physicians OR Doctor OR doctors OR “physician assistant” OR “physician assistants” OR Clinician OR clinicians OR “Medical staff” OR “Medical staffs” OR “Healthcare practitioner” OR “Healthcare practitioners” OR “Healthcare worker” OR “Healthcare workers” OR “Hospital staff” OR “Hospital staffs”) AND title(Well-being OR wellbeing OR “Healthcare workload” OR “Mental health” OR prosperity OR “Worker health” OR “Quality of life” OR “Job satisfaction” OR “Psychological well-being” OR “Compassion satisfaction” OR Wellness OR Self-Care OR “Occupational health” OR “Work environment” OR “Mental stress” OR “Professional burnout” OR Anxiety OR Depression OR Burnout OR “Job stress” OR stress) AND title(tool OR tools OR measurement OR measuring OR measurements OR Instrument OR Instruments) AND (la.exact(“ENG”) AND pd(20140805-20240805))
