# Supplementary material for: Healthcare Professionals’ Subjective Well-Being: A Systematic Review and Methodological Appraisal of Conceptual Models, Measurement Instruments, and Associated Factors
Source: Int J Environ Res Public Health. 2026 Mar 6;23(3):329. doi: 10.3390/ijerph23030329 (PMC13026122; doi:10.3390/ijerph23030329)
Supplement: Supplementary file 1 [file ijerph-23-00329-s001.zip › S4_Conceptual models, Research Instruments, Influencing Factors.pdf]

**Table S5.** Conceptual models within which the well-being of healthcare professionals was studied.

| Model | Description of the Model, Focus of the Study | Dimensions to be Explored by the Model | Number of Studies Assessing | References to Studies |
|---|---|---|---|---|
| Working models of environment and organizational factors | | | | |
| Job demand-resources model | Analyse the balance between work demands and resources that affect burnout and emotional well-being. | • Emotional stress<br>• Workload<br>• Role conflicts<br>• Autonomy<br>• Social support<br>• Professional development<br>• Work engagement Burnout<br>• Well-being | 6 | [53]<br>[25]<br>[50]<br>[61]<br>[46]<br>[28] |
| Quality of Life Theory | Look at quality of life through physical, psychological and social dimensions. | • Physical well-being<br>• Psychological<br>• Well-being Social well-being<br>• Environmental factors | 3 | [41]<br>[33]<br>[43] |
| The job demands-control-support model | Look at the balance between work demands, control and social support and its impact on well-being. | • Job requirements<br>• Autonomy<br>• Social support | 2 | [47]<br>[17] |
| Effort-reward imbalance theory | Analyses the imbalance between workload and pay and its impact on burnout. | • Effort<br>• Salaries<br>• Imbalance between work and pay | 1 | [71] |
| OECD Model of Occupational Well-being | Offers a multidimensional assessment of professional well-being. | • Working conditions<br>• Autonomy<br>• Remuneration<br>• Social support<br>• Professional development<br>• Work-life balance | 1 | [25] |
| Mini-Z model of working life | Investigates the relationship between the quality of working environment and occupational well-being. | • Workload<br>• Autonomy<br>• Management support | 1 | [30] |
| The well-being at work model | Look at the role of workload, support and professional autonomy in well-being. | • Job requirements<br>• Work resources<br>• Psychological well-being | 1 | [60] |
| Stress and burn out models | | | | |
| Maslach burnout model | Focuses on emotional exhaustion, depersonalisation and a diminished sense of professional achievement. | • Emotional exhaustion<br>• Depersonalisation<br>• Sense of personal achievement | 5 | [71]<br>[32]<br>[51]<br>[31]<br>[39] |
| The psychological stress model | Explains stress perception and coping strategies in the work environment. | • Primary and<br>• Secondary<br>• Assessment<br>• Coping<br>• Strategies | 2 | [17]<br>[31] |
| Perceived stress model | Focuses on the subjective perception of stress and the emotional response to external factors. | • Perceived stress coping strategies | 1 | [40] |
| The psychological workload model in healthcare | Analyse the impact of workload on the well-being of health professionals. | • Workload<br>• Administrative workload<br>• Support systems | 1 | [48] |
| Theory of secondary traumatic stress | Explores the emotional strain of dealing with traumatic events. | • Co-survival stress<br>• Emotional exhaustion<br>• Depersonalisation | 1 | [49] |
| Psychological and emotional well-being models | | | | |
| Self-Determination Theory | Emphasis's autonomy, competence and social belonging as the basis for emotional well-being. | • Autonomy<br>• Competences<br>• Social belonging | 4 | [46]<br>[38]<br>[34] |
| The psychological well-being model | Explains psychological well-being as multidimensi onal phenomenon. | • Autonomy<br>• Personal<br>• development<br>• Social relationships<br>• Meaning of life<br>• Self-acceptance<br>• Environmental control | 1 | [67] |
| Resilience theory | Focuses on psychological resilience, emotional stability and social support. | • Psychological resilience<br>• Social support<br>• Optimism<br>• Self-efficacy | 1 | [34] |

| | | | | |
|---|---|---|---|---|
| Health promotion model | Focuses on individual health behaviors and their impact on emotional well-being. | • Personal characteristic<br>• Emotional factors<br>• Environmental influences | 1 | [64] |
| Positive mental health model | Look at the impact of positive emotional and psychological well-being on the work environment. | • Emotional well-being<br>• Psychological well-being<br>• Social well-being | 1 | [52] |
| Social and emotional support models | | | | |
| Quality of Working Life Model | Assesses the quality of professional life, mpassion, satisfaction and fatigue. | • Compassion<br>• Satisfaction Fatigue<br>• Secondary traumatisation | 1 | [59] |
| Emotional labour theory [48] | Explores the impact of emotional labour on occupational well-being and the risk of burnout. | • Emotional work<br>• Superficil work<br>• Deep work | 1 | [63] |
| Models of positive psychology and well-being | | | | |
| The theory of subjective well-being | Focuses on emotional response, life satisfaction and the balance between positive and negative emotions. | • Emotional reactions<br>• Life satisfaction<br>• Balance of positive and negative emotions | 4 | [43]<br>[40]<br>[26]<br>[70] |
| World Health Organisation Well-being Index | Use subjective well-being measurement to identify emotional health risks. | • Joy<br>• Relaxation<br>• Energy<br>• Interest in life Sufficient rest | 1 | [25] |
| Positive psychology approaches | Promotes the importance of positive emotions, psychological resilience and personal growth. | • Positive emotions<br>• Personal growth<br>• Psychological resilience | 1 | [23] |
| Physician well-being index | Assesses the well-being of doctors by identifying professional risks. | • Burnout<br>• Stress<br>• Job satisfaction | 1 | [56] |
| Organisational culture and management models | | | | |
| Competing Values Framework | Analysis of organisational management strategies on well-being. | • Flexibility and control<br>• Results orientation<br>• Relationship building | 1 | [58] |
| Psychosocial model | Explain the psychosocial factors that influence well-being and health in the work environment. | • Job requirements<br>• Psychosocial risks<br>• Work environment factors | 1 | [21] |
| Resource Conservation Theory | Explores the role of resource conservation in emotional resilience and burnout prevention. | • Conservation of resources<br>• Resource accumulation<br>• Loss of resources | 1 | [61] |
| Employee Engagement Theory | Explain the impact of employee engagement and motivation on wellbeing and performance. | • Physical emotional<br>• Cognitive engagement | 1 | [19] |

**Table S6.** Research Instruments Used in the Study of Healthcare Professionals' Subjective Well-Being.

| Category | Instruments | References |
|---|---|---|
| Subjective Well-Being and Positive Mental Health Scales | Multiple Happiness Questionnaire (MHQ) | [63] |
| | Subjective Well-being Scale (SWS) | [64] |
| | WHO-5 Well-Being Index | [65] |
| | Satisfaction with Life Scale (SWLS) | [66] |
| | Psychological Well-Being Scale (Ryff) | [39] |
| | Mental Health Continuum-Short Form (MHC-SF) | [44] |
| | Subjective Happiness Scale (SHS) | [67] |
| | Positive Mental Health (PMH) instrument | [45] |
| | Brief Resilience Scale (BRS) | [68] |
| Burnout | Maslach Burnout Inventory (MBI) | [24] |
| | Oldenburg Burnout Inventory (OLBI) | [69] |
| | Burnout Assessment Tool (BAT) | [70] |
| | Work Ability Index | [71] |
| | Stanford Professional Fulfillment Index (PFI) | [10] |
| Psychological Distress and Disorder | Patient Health Questionnaire-9 (PHQ-9) | [72] |
| | Generalized Anxiety Disorder-7 (GAD-7) | [73] |
| | Self-rating Depression Scale (SDS) | [74] |
| | Self-rating Anxiety Scale (SAS) | [75] |
| | Depression, Anxiety, and Stress Scale (DASS-21) | [76] |
| | PTSD Checklist (PCL-5) | [77] |
| Psychosocial Stress and Workload | Perceived Stress Scale (PSS) | [30] |
| | Effort-Reward Imbalance Scale | [15] |
| | Fear of COVID-19 Scale (FCV-19S) | [78] |
| | NASA Task Load Index (NASA-TLX) | [79] |
| | Emotional Distress Scale | [80] |
| Aspects of Work Engagement and Well-Being | Leiden Quality of Work Questionnaire for Medical Doctors | [81] |
| | Job Crafting Scale (JCS) | [82] |

| | | |
|---|---|---|
| | Utrecht Work Engagement Scale (UWES) | [83] |
| | Workplace Well-Being Scale (WWB) | [84] |
| Sleep quality | Pittsburgh Sleep Quality Index (PSQI) | [85] |
| | Insomnia Severity Index | [86,87] |
| | Pittsburgh Sleep Quality Index | [85] |
| Social support, Interpersonal communication | Multidimensional Scale of Perceived Social Support (MSPSS) | [88] |
| | Interpersonal Reactivity Index (IRI) | [89] |
| Resilience, personal development | Connor-Davidson Resilience Scale (CD-RISC) | [41] |
| | Resilience Scale-13 (RS-13) | [90] |
| | Dispositional Hope Scale | [91] |
| | Comprehensive Relative Autonomy Index (CRAI) | [92] |
| Working environment, Working conditions | Kurzfragebogen zur Arbeitsanalyse (KFZA) | [93] |
| | Copenhagen Psychosocial Questionnaire (COPSOQ) | [94] |
| | WHO PPE Assessment Tool | [95] |
| | Health and Safety Executive Management Standards Indicator Tool (HSE-MS IT) | [96] |

**Table S7.** Factors influencing the subjective well-being of healthcare professionals.

| Factors | Definition | Reference of Definition | Number of Studies | Number of the Study in Table S2 |
|---|---|---|---|---|
| | Psychological factors | | | |
| Depression | Persistent depressed mood, loss of interest in daily activities, fatigue and difficulty concentrating. | [117] | 17 | 5, 6, 7, 10, 12, 15, 17, 18, 20, 21,25, 26,28, 31, 32, 35, 38 |
| Anxiety | Increased anxiety, fear and tension, which can manifest at both cognitive and physiological levels. | [118] | 14 | 6, 7, 10, 12, 15, 18, 20, 21,25, 26, 28, 31, 32, 36 |
| Stress | A natural reaction to demands and pressures that can cause physical and psychological health problems over a long period of time. | [119] | 11 | 3, 9, 10, 14, 20, 21, 27, 32, 36, 39, 40 |
| Burnout | The result of prolonged work stress, including emotional exhaustion, depersonalisation and reduced personal effectiveness. | [77] | 10 | 8, 20, 23,28, 31, 34, 36, 40, 42, 48 |
| Life satisfaction | An individual's subjective assessment of their own quality of life and well-being. | [83] | 5 | 2, 14, 23, 29, 36 |
| Mental health | An individual's ability to function effectively, maintain emotional balance and adapt to challenges. | [81] | 3 | 14, 23, 26 |
| Mental resilience | The ability to overcome difficulties and maintain emotional stability in the long term. | [120] | 2 | 7, 22 |
| Compassion fatigue | A state of emotional exhaustion caused by prolonged involvement in alleviating the suffering of others. | [121] | 2 | 31, 34 |
| Positive emotions | Positive emotions such as joy, satisfaction and gratitude, which contribute to psychological well-being. | [122] | 2 | 2, 3 |
| Psychological distress | Negative emotional reactions to prolonged stress and overload. | [123] | 2 | 10, 12 |
| Stress resilience | The individual's ability to manage stress effectively and maintain balance in pressure situations. | [124] | 2 | 36, 48 |
| Suicidal thoughts | Thinking about suicide as a possible solution to life's difficulties. | [125] | 1 | 17 |
| Irritability | Increased intolerance and anger, often linked to stress. | [126] | 1 | 28 |
| Subjective well-being | The sum of an individual's emotional well-being, positive emotions and life satisfaction. | [90] | 1 | 3 |
| Negative emotions | Anger, sadness or frustration negatively affects well-being. | [127] | 1 | 3 |
| Nervousness | Feelings of anxiety and tension that affect cognitive and behavioural responses. | [128] | 1 | 10 |
| Tension | A physical and emotional response to prolonged stress. | [129] | 1 | 10 |
| Empathy | The ability to understand and empathise with the emotions of others. | [130] | 1 | 22 |
| Fatigue | Feeling exhausted, affecting energy levels and work capacity. | [131] | 1 | 10 |
| Life vitality | An individual's self-image and ability to evaluate their own achievements. | [132] | 1 | 2 |
| Self-worth | An individual's overall sense of value and personal worth. It reflects the belief that one is a good person who deserves love and respect, regardless of achievements or others' evaluations. | [133] | 1 | 22 |
| Altruistic behaviour | Altruistic behaviour is a selfless concern for the well-being of others, often involving helping actions without expecting personal benefit. | [134] | 1 | 2 |
| Personal growth | An individual's active process of realizing their potential and striving for self-improvement and psychological development | [135] | 1 | 2 |
| Mindfulness | Mindfulness is the awareness that emerges through paying attention on purpose, in the present moment, and non-judgmentally to the unfolding of experience. | [136] | 1 | 3 |

| | | | | |
|---|---|---|---|---|
| Mental protection | The sum of an individual's emotional well-being, positive emotions and life satisfaction. | [129] | 1 | 23 |
| Emotional stability | Ability to maintain emotional balance in stressful situations. | [137] | 1 | 29 |
| Self-stigma (help-seeking behavssiour) | Self-stigma refers to the internalized shame and negative beliefs individuals hold about themselves for seeking psychological help, often leading to reduced help-seeking behavior. | [138] | 1 | 31 |
| | Work environment factors | | | |
| Working environment, conditions | Physical and psychological conditions in the workplace affect employee well-being and performance. | [139] | 8 | 7, 10, 25, 27, 31, 39, 41, 45 |
| Workload | The amount and intensity of work, which can cause stress and affect the quality of work. | [107] | 5 | 6, 19, 23, 26, 28, |
| Work involvement | Employee engagement and motivation to perform their duties with enthusiasm and energy. | [108] | 4 | 10, 23, 27, 48 |
| Work-life balance | Work–life balance, defined as the equilibrium between work responsibilities and personal life, is a critical factor influencing employee well-being and job satisfaction | [140] | 4 | 9, 10, 23, 32 |
| Job satisfaction | The employee's subjective assessment of the job and its relevance to his/her wants and needs. | [141] | 4 | 10, 14, 39, 48 |
| Work control | The employee's ability to influence decisions and determine how work is done. | [142] | 3 | 11, 25, 48 |
| Job Demands | The physical and psychological demands that the work environment and tasks place on the employee. | [75] | 3 | 11, 25, 48 |
| Infectious diseases | Infectious diseases are disorders caused by organisms such as bacteria, viruses, fungi or parasites that can spread, directly or indirectly, from one individual to another, posing occupational risks to healthcare workers through contact with patients, bodily fluids, or contaminated environments. | [143] | 3 | 18, 19, 26 |
| Management support | Management support refers to the actions and behaviors of supervisors and organizational leaders that provide guidance, resources, and emotional support to employees, contributing to their well-being, job satisfaction, and performance. | [144] | 2 | 25, 31 |
| Motivation | Internal and external factors that contribute to an employee's desire to achieve professional goals. | [145] | 2 | 33, 48 |
| Professional development opportunities | Professional development opportunities—defined as structured programs for enhancing employee skills, knowledge, and career growth—have been shown to significantly improve performance outcomes in educational contexts. | [146] | 2 | 20, 42 |
| Communication with management | Effective communication between management and staff, ensuring clarity and transparency. | [147] | 2 | 10, 19 |
| Patient mortality | Patient mortality in healthcare refers not only to the event of patient death but also encompasses the emotional and psychological impact it has on healthcare staff, including grief, stress, and moral distress. | [148] | 2 | 7, 10 |
| Feedback | Feedback in the workplace refers to the information provided by supervisors or peers regarding an employee's performance, aimed at reinforcing effective behaviors and guiding improvement. | [149] | 1 | 10 |
| Autonomy | Autonomy refers to the degree to which a job provides an employee with substantial freedom, independence, and discretion in scheduling the work and determining the procedures to carry it out. | [150] | 1 | 33 |
| Remuneration | Remuneration refers to the compensation employees receive for their work, including salary, benefits, and incentives, and its perceived fairness relative to job demands and responsibilities. | [151] | 1 | 7 |
| Meaningful work | Meaningful work refers to the perception that one's job has personal significance, purpose, and contributes to something beyond the self. | [152] | 1 | 22 |
| Work resources | Work resources refer to the physical, psychological, social, or organizational assets that help employees achieve work goals, reduce job demands, and stimulate personal growth and development. | [153] | 1 | 9 |
| Communication with colleagues | Communication with colleagues refers to the exchange of information, feedback, and support among team members that enhances coordination, reduces misunderstandings, and fosters a collaborative work climate. | [154] | 1 | 9 |
| Willingness to work in the healthcare sector | individual's sustained motivation and intention to remain employed in healthcare, driven by intrinsic goals such as purpose, competence, and autonomy. | [155] | 1 | 8 |
| Time limit for the task to be accomplished | Time limit for the task to be accomplished refers to the constraints imposed by deadlines, which create pressure to complete tasks within a specific time frame. These constraints can influence performance, stress levels, and team dynamics. | [156] | 1 | 9 |
| Verbal and physical violence | Verbal and physical violence refers to incidents where workers are exposed to aggressive behavior—either verbal threats, insults, or physical assaults—within the workplace. Such experiences can negatively affect employees' psychological well-being, job satisfaction, and safety perception. | [157] | 1 | 7 |

| | | | | |
|---|---|---|---|---|
| Individual factors | | | | |
| Mental health | Mental health is defined as a state of well-being in which an individual realizes their abilities, can cope with the normal stresses of life, can work productively, and is able to contribute to their community. It includes the ability to manage stress, maintain emotional balance, and function effectively in everyday life. | [158] | 5 | 14, 23, 26, 31, 38, |
| Quality of sleep | Quality of sleep refers to the duration, continuity, depth, and restorative properties of sleep, which are essential for maintaining physical health, cognitive functioning, and emotional regulation. | [159] | 4 | 1, 5, 12, 26 |
| Physical health | Physical health refers to the overall condition of the body and its ability to perform daily activities without physical limitations or illness. It encompasses physical functioning, absence of disease, energy levels, and fitness. | [160] | 4 | 28, 31, 33, 38 |
| Nutrition | Nutrition refers to dietary habits and nutrient intake that significantly influence physical health, energy levels, cognitive performance, and mental well-being. | [161] | 2 | 1, 5 |
| Fatigue | Fatigue is a feeling of exhaustion often linked to workload, disturbed sleep, and stress, adversely affecting both physical and mental performance. | [162] | 2 | 5, 10 |
| Quality of life | Quality of life refers to an individual's perception of their position in life—within the context of cultural values, personal goals, expectations, and concerns. It encompasses physical health, psychological state, level of independence, social relationships, and connection to salient features of the environment | [163] | 2 | 15, 16 |
| Physical activity | Physical activity refers to any bodily movement produced by skeletal muscles that substantially improves physical health, energy levels, and mental well-being when performed frequently | [164] | 1 | 5 |
| Self-rated well-being | An individual's self-assessment of his or her well-being, including physical and emotional well-being. | [78] | 1 | 5 |
| Loss of pleasure in daily activities | Loss of pleasure in daily activities, also known as anhedonia, refers to a markedly reduced capacity to experience enjoyment or interest in routine activities, commonly associated with depressive symptoms and burnout. | [165] | 1 | 10 |
| Meeting basic needs | Providing basic daily needs such as food, sleep and safety. | [166] | 1 | 14 |
| Personality traits | Personality traits refer to enduring patterns of thoughts, feelings, and behaviors that distinguish individuals and influence their attitudes toward work, coping with stress, and social relationships. The Five-Factor Model (FFM) includes five core traits: neuroticism, extraversion, openness, agreeableness, and conscientiousness.. | [167] | 1 | 36 |
| Social factors | | | | |
| Social support | The emotional, instrumental and informational support an individual receives from other people. | [93] | 6 | 1, 10, 11, 33, 38, 39 |
| Support from family and friends | Support from family and friends refers to emotional and practical assistance provided by close social relationships, such as family members and friends. This type of support plays a key role in promoting psychological well-being and buffering the negative effects of stress. | [111] | 1 | 26 |
| Involvement in decision-making | Involvement in decision-making refers to the extent to which employees are allowed or encouraged to participate in decisions that impact their tasks or work environment. Greater involvement is associated with increased motivation, job satisfaction, and organizational commitment. | [168] | 1 | 10 |
| Social stigma (social pressure and stigmatisation of health workers) | Social stigma, in the context of healthcare, refers to the negative societal stereotypes, discrimination, and social pressures faced by health workers, especially in relation to mental health or crisis exposure. This can undermine their well-being, professional identity, and willingness to seek support. | [169] | 1 | 19 |
| Demographic factors | | | | |
| Age | Age refers to the number of years an individual has lived and is a fundamental biological and demographic factor that can influence physical health, psychological well-being, stress vulnerability, and work performance in healthcare settings. | [170] | 48 | 1-48 |
| Gender | Gender refers to the socially constructed roles, behaviors, expressions, and identities of individuals as men, women, or gender-diverse persons. | [171] | 48 | 1-48 |
| Marital status | Marital status refers to whether an individual is single, married, divorced, or widowed. This status can influence levels of social support, emotional stability, and work-life balance, thereby impacting psychological well-being and health outcomes. | [172] | 6 | 6, 7, 14, 16,17, 32 |
| Education | Education refers to the level of formal academic and professional training a person has attained | [173] | 5 | 6, 7, 14, 16, 17 |

| | | | | |
|---|---|---|---|---|
| Specialty | Specialty refers to the specific medical or healthcare field (e.g., surgery, psychiatry, emergency medicine) in which a professional works. | [174] | 5 | 6, 7, 10, 16, 17 |
| Work experience in healthcare | The length of experience of the employee in healthcare. | [175] | 4 | 6, 14, 16, 17 |
